# Sequence-based predictive modeling to identify cancerlectins

**DOI:** 10.18632/oncotarget.15963

**Published:** 2017-03-07

**Authors:** Hong-Yan Lai, Xin-Xin Chen, Wei Chen, Hua Tang, Hao Lin

**Affiliations:** ^1^ Key Laboratory for Neuro-Information of Ministry of Education, School of Life Science and Technology, Center for Informational Biology, University of Electronic Science and Technology of China, Chengdu, China; ^2^ Department of Physics, School of Sciences, and Center for Genomics and Computational Biology, North China University of Science and Technology, Tangshan, Tangshan, China; ^3^ Department of Pathophysiology, Southwest Medical University, Luzhou, China

**Keywords:** cancerlectins, binomial distribution, optimal tripeptides, SVM

## Abstract

Lectins are a diverse type of glycoproteins or carbohydrate-binding proteins that have a wide distribution to various species. They can specially identify and exclusively bind to a certain kind of saccharide groups. Cancerlectins are a group of lectins that are closely related to cancer and play a major role in the initiation, survival, growth, metastasis and spread of tumor. Several computational methods have emerged to discriminate cancerlectins from non-cancerlectins, which promote the study on pathogenic mechanisms and clinical treatment of cancer. However, the predictive accuracies of most of these techniques are very limited. In this work, by constructing a benchmark dataset based on the CancerLectinDB database, a new amino acid sequence-based strategy for feature description was developed, and then the binomial distribution was applied to screen the optimal feature set. Ultimately, an SVM-based predictor was performed to distinguish cancerlectins from non-cancerlectins, and achieved an accuracy of 77.48% with AUC of 85.52% in jackknife cross-validation. The results revealed that our prediction model could perform better comparing with published predictive tools.

## INTRODUCTION

Lectins are highly specific proteins which have more than one carbohydrate-binding site and are typically able to agglutinate certain animal cells and/or precipitate glycoconjugates [1, 2]. It should be noted that lectins differ greatly from antibodies, although some antibodies bind to antigens and cause agglutination reaction in a similar way to lectins. Up to present, almost all organisms, including viruses, bacteria, plants, vertebrates, invertebrates have been found to be able to synthesize and secrete lectins [3]. It has also been revealed that lectins are involved in a wide variety of biological processes, e.g., the growth, differentiation and development of cells, cell adhesion and migration, the interaction between cell and extracellular matrix, apoptosis, the modulation of immune defense and inflammatory response [4–6]. Accordingly, numerous researches in many fields of cell biology, biochemistry, as well as immunology often utilize lectins as diagnostic and therapeutic tools [7].

Cancerlectins are a group of lectins that are inseparably linked with cancer and known to play various important roles in cancer initiation, survival, growth, metastasis and spread [8–11]. They have been widely applied in cancer study from fundamental research to clinical application [12]. For instance, sialic acid-binding immunoglobulin-type lectins-9 (Siglecs-9), which demonstrates neutrophilic granulocyte specific expression, can bind to the glycans presenting on tumor cell surfaces and regulate immune response, and then facilitate or inhibit cancer progression [13]. A wide array of studies have indicated that cancerlectins can be considered as diagnostic and molecularly therapeutic markers for tumor, or as molecular tools of cancer prevention and prognosis [1, 14, 15]. Therefore, it's significant to screen the particular cancerlectins from multitudinous lectins for better understanding and even conquering cancer.

Experimental assays have identified and functionally annotated a lot of cancerlectins, overwhelming majority of which are archived and integrated in the database of CancerLectinDB [16]. These experimental detections of cancerlectins are extremely accurate and reliable. Nevertheless, they are customarily low-efficiency and high-cost. With the continuously rapid advancement of sequencing technology, more and more cancerlectin protein sequences are stored, and the computational prediction of cancerlectins emerges naturally. Thus, various models have been proposed to identify cancerlectins based on the sequence similarity, amino acid or dipeptide composition and evolutionary information of these cancerlectins [17–19]. Machine learning techniques such as Support Vector Machine (SVM) [20, 21], Artificial Neural Network (ANN), decision tree and random forest have been utilized to perform classification and regression [17–19, 22–24]. However, the predictive power of the above-mentioned methods is limited and the accuracies are not particularly high. There is still much room for improvement in prediction accuracy, hence, this work puts forward a novel feature extraction method, which has stronger capability of predicting in some degree.

## RESULTS AND DISCUSSION

### Prediction performance

As described in the section of feature description, each sample lectin sequence was translated into a vector of 8000 over-represented tripeptides. Using too many features with low confidence level to train a predictive model will be relatively time-consuming and have a strong likelihood of establishing an overfitting model. On the contrary, if the number of feature tripeptides is too small, they will not afford enough information. They can only describe part of cancerlectin properties even though every one of them may have a high confidence level and be extremely informative. Both of these two situations will result in poor prediction [25]. For example, 6594 tripeptides with > 50% confidence level produced an accuracy of 64.6% for identifying cancerlectins. Similarly, by using > 99.99% as the confidence level, we obtained the top 53 tripeptides, but the overall accuracy was only 67.08% in 7-fold cross-validation. Thus, it is crucially important to choose an appropriate number of features for the construction of a robust and efficient prediction model.

**Figure 1:**
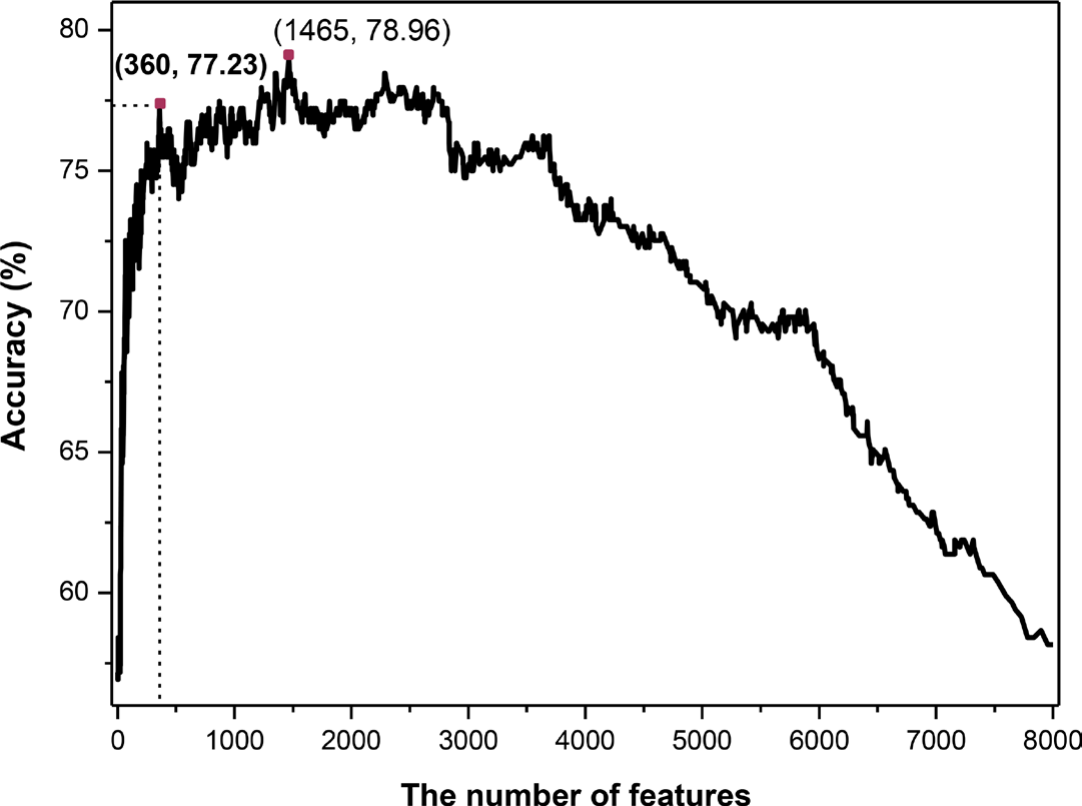
The 7-fold cross-validated accuracies of different predictive models constructed with different number of features

On the basis of binomial distribution, a novel feature selection technology was proposed in this work (see in section of Method). Then the SVM classifier was employed. The 7-fold cross-validated results (Figure 1) showed that the maximum overall accuracy of 78.96% was achieved when the top 1465 tripeptides was used. However, the total number of sample proteins is 404, which is much less than the number of feature dimension. For the purpose of establishing a credible and robust model, we should take into the number of features and the accuracy simultaneously. Ultimately, we chose the top 360 tripeptides which could produce an overall accuracy of 77.23% which was just slightly lower than the maximum accuracy (78.96%) produced by the top 1465 features. Therefore, the 360 tripeptide compositions served as the optimal feature subset to construct the final classifier in this study.

**Figure 2:**
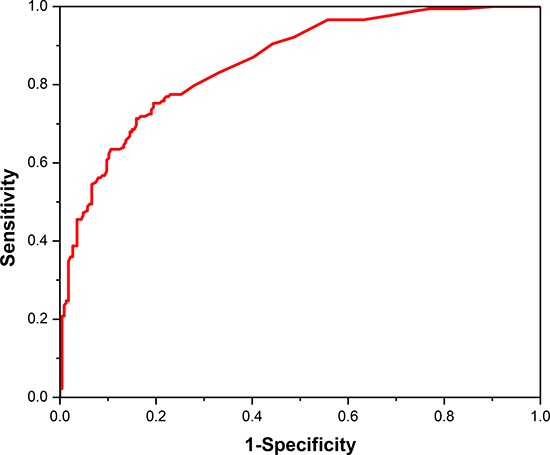
The ROC curve for cancerlectin prediction using the optimal 360 tripeptides

The jackknife cross-validation was conducted for performance assessment owing to the imbalance between positive dataset and negative dataset. And the final values of the two SVM parameters are *c* = 2^11^ and *g* = 2^−13^. The statistical analysis indicated that our predictor have a relatively excellent prediction performance with an overall accuracy of 77.48%. And the sensitivity and specificity of the proposed model are 75.28% and 80.53%, respectively. We also draw the ROC curve in Figure 2. It shows that the AUC reaches to 0.855, suggesting an excellent prediction capability of our model.

### Comparison with existing methods

To estimate whether a novel prediction model is good enough, it is necessary to compare it with other published methods. In the past, some computational models have been developed using diverse methods based on the same sample dataset. The comparison results were recorded in Table 1. Kumar et al. firstly developed prediction models using amino acid compositions, dipeptide compositions, split compositions, evolutionary and domain information. The analytic results showed that the SVM model based on the integration of PROSITE domain information and position specific scoring matrix(PSSM) achieved the maximum accuracy of 69.09% [17]. Lin et al. developed a model to predict cancerlectins by g-gap dipeptides and obtained a higher accuracy of 75.19% [18]. Our lectin sequence-based predictive modeling can identify cancerlectins with the highest accuracy, sensitivity and specificity of 77.48%, 75.28%, 80.53%, respectively. These comparison results indicate that our new predictor is more powerful in discriminating cancerlectins from non-cancerlectins.

**Table 1:** Performances of various existing predictive models

Methods	*S*_n_ (%)	*S*_p_ (%)	*Acc* (%)
**Our predictor**	**75.28**	**80.53**	**77.48**
Lin et al. [18]	69.10	80.10	75.19
Kumar et al. [17]	68.00	69.90	69.09

## MATERIALS AND METHODS

A positive dataset containing 385 experimentally validated and non-duplicated cancerlectin sequences was collected from the work of Lin [18], and the raw data of which was downloaded from the CancerLectinDB [16]. This database has retrieved cancer-related lectins and their corresponding sequence, structure, function information from literatures [16]. The application of the keyword “lectin” in searching the UniProt database (http://www.uniprot.org/) created the negative samples, which consisted of 820 proteins after eliminating sequences labeled with “similar”, “fragment”, “putative”, “probable”. In order to get rid of the influence of the data redundancy on prediction results, the CD-HIT program [26] was applied to filter the highly similar samples by setting 50% as cut-off. Consequently, we gained a total of 178 cancerlectin and 226 non-cancerlectin sequences.

### Feature description

A comprehensive review of protein attribute prediction [27] stated that besides a reliable and objective benchmark protein sequence dataset, the perfect formulation of protein sample is necessary for the development of a high-throughput automated predictive tool. The simplest and also most popular approach to formulate protein sequences is amino acid composition (AAC) [28, 29] which uses the normalized frequency of each amino acid in one protein sample. The conjoint triad feature [19, 28] encodes each protein sequence by using a triad frequency distribution. In present research, in order to get the sequence-order information, the adjacent tripeptide composition was used instead of the classical AAC to represent a protein sample. A variety of proteins in an organism are made up of 20 standard amino acids, hence there are total 20 × 20 × 20 = 8000 possible tripeptides. Thus, we transformed a cancerlectin or non-cancerlectin protein sample *P* with *L* amino acids into an input vector of 8000 dimensions, *F*_8000_, defined as follows.

$$F_{8000}=\;{\left[f_{1},\;f_{2},\;\ldots ,\;f_{i},\;\ldots ,\;f_{8000}\right]}^{T}$$ (1)

In Eq. (1), the symbol *T* represents the transposition of a vector and *f_i_* is the frequency of the *i*-th tripeptide appearing in a cancerlectin or non-cancerlectin sequence. These frequencies can be calculated using the formula (2),

$$f_{i}=\frac{n_{i}}{\sum_{i=1}^{8000}n_{i}}=\;\frac{n_{i}}{L-2}$$ (2)

where *n_i_* means the number of occurrences of the *i*-th tripeptide in a protein composed of *L* amino acid residues.

### The optimal feature subset selection

The feature set contains 8000 features, which may lead to the curse of dimensionality. This large feature set undoubtedly contains some redundant or irrelevant features and those should be excluded for improving efficiency and robustness of model. It is worth picking out those relevant features which are the most useful for prediction model construction. The optimal feature subset will shorten the training and utilization times, reduce the measurement and storage requirements, avert overfitting and improve prediction performance [30]. Up to date, many effective feature selection techniques such as the analysis of variance [31], max-relevance-max-distance [32], minimum redundancy maximum relevance [33], principal component analysis [34] and recursive feature elimination algorithm [35, 36] have been proposed to reduce effects from noise or irrelevant features and provided good prediction results.

In current study, we introduced a new feature selection technique based on binomial distribution to screen the informative tripeptides [37, 38]. In order to judge whether it is an essentially random event that one certain tripeptide occurs in one kind of protein, first, we calculated the prior probability *q_j_* which is formulated with the form of Eq. (3).

$$q_{j}=\frac{m_{j}}{M}$$ (3)

where *m_j_* represents the number of tripeptides in the j-th category of sample, where *j* = 1, 2 and *M* is the total occurrence number of all tripeptides contained in the both positive and negative data sets.

Second, we calculated the probability *P*(*n_ij_*) of the *i*-th tripeptide occurring in the *j*-th category of sample *n_ij_* or more times by using Eq. (4)

p(nij)=∑m=nijNiNi!m!(Ni−m)qj(1−qj)mNi−m (4)

where the sum in Eq. (4) is taken from *n_ij_* to *N_i_*. The total occurrence numbers of a given *i*-th tripeptide in the *j*-th class of protein and in the benchmark dataset are denoted by *n_ij_* and *N_i_*, respectively. If the *i*-th tripeptide occurring in the *j*-th category of protein is not random and biologically significant, the *P*(*n_ij_*) will be very small. Thus, we may define the confidence level of this statement as *CL_ij_*:

$$\;CL_{ij}=1-\;P\left(n_{ij}\right)$$ (5)

Accordingly, each of the 8000 tripeptides has two *CL* values because of the two kinds of proteins considered in this work. Then, we assigned the lager one to be the *CL* of the *i*-th tripeptide, like this:

$$CL_{i}=max(\;CL_{i1},\;\;CL_{i2})$$ (6)

Then the feature subsets were listed in descending order according to their *CLs*.

Finally, we applied the incremental feature selection strategy to determine the optimal number of feature subset, the process of which is described as follows: the first feature subset was composed of the tripeptides with the biggest *CL* value, followed by the producing of a new feature subset by adding the tripeptides with the second biggest *CL* value into the first feature subset. Repeating the aforementioned second step from higher *CL* values to lower *CL* values until all the candidate tripeptides were added. Consequently, for every newly generated feature subset, a predictive model was trained on the basis of SVM and was assessed by 7-fold cross-validation. The optimal feature subset can be picked out according to its maximum prediction accuracy.

### Support vector machine

The support vector machine (SVM) was invented by Vapnik et al. based on the study of statistical learning theory [39]. In the field of machine learning, SVM is a supervised learning model and is usually used for pattern recognition, classification and regression analysis. The basic idea of SVM is: 1) to transform the non-linearly separable sample data, namely the input sample space, into a new feature space which is a high-dimension and linearly separable Hilbert space via a non-linear mapping; 2) to construct an optimal hyperplane which maximizes the degree of separation between the two classes. One of the potential advantages of SVM is that it is still effective in cases that the number of features is greater than that of samples. Additionally, SVM is versatile that different Kernel functions can be specified for the decision function. Thus, the classifier has been widely applied to solve bioinformatics problems, e.g., identification of bacterial secreted proteins, recognition of phosphothreonine sites in human proteins, prediction of microRNA targets, classification of disease drugs, identification of tumor subtypes, and so forth [40–43]. In this study, we utilized the LibSVM 3.21 software which can be freely downloaded from https://www.csie.ntu.edu.tw/~cjlin/libsvm/ to implement SVM to discriminate cancerlectins from non-cancerlectins. The two pivotal parameters for SVM, the regularization parameter *c* and the kernel width parameter *g*, were optimized using grid search based on cross-validation test. The search spaces of these were [215, 2–5] and [2–5, 2–15] with steps being 2 and 2^−1^, respectively.

### Performance assessment

In statistical prediction, various cross-validation methods are generally utilized for evaluating the performance of a predictor, such as independent dataset test, subsampling test and jackknife cross-validation test [27]. Jackknife cross-validation can always yields a unique outcome for a given benchmark dataset [44, 45]. Generally, jackknife test has two evident advantages: 1) the estimated generalization error is more reliable, because in each iterative process of jackknife test, almost all of the samples are used to train the model; 2) in the testing procedure, no random factors will affect the testing data and ensure that testing procedure can reproduce. Thus, the jackknife cross-validation was used to examine the performance of final model.

The performance of classification between cancerlectins and non-cancerlectins was evaluated by three indexes, including accuracy (*Acc*), sensitivity (*S_n_*) and specificity (*S_p_*). *Acc* is the overall accuracy of the discrimination between cancerlectins and non-cancerlectins. *S_n_* and *S_p_* reflect the sensitivity and specificity of the SVM prediction model, which mean the ability to correctly identify cancerlectins and correctly recognize non-cancerlectins, respectively. The general formulations of these measures are as following:

$$Acc=\frac{TP+TN}{TP+TN+FP+FN}$$ (7)

$$S_{n}=\;\frac{TP}{TP+FN}$$ (8)

$$S_{p}=\;\frac{TN}{TN+FP}$$ (9)

In the above formulas, *TP* (Ture positive) and *TN* (Ture negative) denote the numbers of correctly predicted cancerlectins and non-cancerlectins, respectively. And, *FP* (False positive) and *FN* (False negative) are the number of known non-cancerlectins but predicted as cancerlectins and the number of known cancerlectins but predicted as non-cancerlectins, respectively. We further plotted a receiver operating characteristic (ROC) curve by using sensitivity as the X-axis and 1-specificity as Y-axis. The value of the area under ROC curve (AUC) is useful for assessing the performance of model across the entire range of decision values.

## CONCLUSIONS

More and more researchers have focused on the roles of cancerlectins or the microarray profiling of them in the prevention, detection, therapy and diagnosis of various human cancers such as breast cancer, pancreatic cancer, hepatocellular carcinoma [46–48]. Therefore, it is significant to recognize cancerlectins. On the basis of the consideration that there is still much room for improvement in prediction accuracy, we designed a predicted model based on optimal tripeptide composition statistically obtained by binomial distribution to improve prediction accuracy. Although the new method got better results for distinguishing cancerlectins from non-cancerlectins when comparing with the existing predictors, the accuracy is still far from satisfactory.

In the future, we shall seek and gather more cancerlectin data and update the benchmark sample set. Furthermore, some powerful and flexible DNA\RNA or protein sequence analysis tools based on the concept of pseudo nucleotide or amino acid composition [49–52] may inspire us to develop another ensemble learning approach. It shall also consider the physicochemical properties, secondary structures and other characteristics of lectins which may contribute to improving the accuracy of distinguishing between cancerlectins and non-cancerlectins. In order to improve the efficiency of applying a new prediction/classification method, a user-friendly and publicly accessible web-server is often established [53–57]. Therefore, we will also make efforts to provide a flexible web-server for that method which may bring some convenience for the vast majority of experiment scientists and medical researchers.
